# High Levels of Class I Major Histocompatibility Complex mRNA Are Present in Epstein–Barr Virus-Associated Gastric Adenocarcinomas

**DOI:** 10.3390/cells9020499

**Published:** 2020-02-21

**Authors:** Farhad Ghasemi, Steven F. Gameiro, Tanner M. Tessier, Allison H. Maciver, Joe S. Mymryk

**Affiliations:** 1 Department of Surgery, Western University, London, ON N6A 3K7, Canada; fghasemi2019@meds.uwo.ca; 2 Department of Microbiology and Immunology, Western University, London, ON N6A 3K7, Canada; sgameiro@uwo.ca (S.F.G.); ttessie2@uwo.ca (T.M.T.); 3 Department of Surgery and Oncology, Western University, London, ON N6A 3K7, Canada; Allison.Maciver@lhsc.on.ca; 4 Department of Microbiology & Immunology, Oncology and Otolaryngology, Head & Neck Surgery, The Western University, London, ON N6A 3K7, Canada; 5 London Regional Cancer Program, Lawson Health Research Institute, London, ON N6C 2R5, Canada

**Keywords:** Epstein–Barr virus, EBV, MHC-I, major histocompatibility complex, antigen presentation, immune evasion, stomach adenocarcinoma, TCGA, EBVaGC

## Abstract

Epstein–Barr virus (EBV) is responsible for approximately 9% of stomach adenocarcinomas. EBV-encoded microRNAs have been reported as reducing the function of the class I major histocompatibility complex (MHC-I) antigen presentation apparatus, which could allow infected cells to evade adaptive immune responses. Using data from nearly 400 human gastric carcinomas (GCs), we assessed the impact of EBV on MHC-I heavy and light chain mRNA levels, as well as multiple other components essential for antigen processing and presentation. Unexpectedly, mRNA levels of these genes were as high, or higher, in EBV-associated gastric carcinomas (EBVaGCs) compared to normal control tissues or other GC subtypes. This coordinated upregulation could have been a consequence of the higher intratumoral levels of interferon γ in EBVaGCs, which correlated with signatures of increased infiltration by T and natural killer (NK) cells. These results indicate that EBV-encoded products do not effectively reduce mRNA levels of the MHC-I antigen presentation apparatus in human GCs.

## 1. Introduction

The anti-viral immune response is composed of several layers of defenses that cooperatively contribute to blocking, controlling, and eliminating infection. Intrinsic and innate immunity serve as the first levels of response and comprise pre-existing host defenses against viral infection [1,2]. Subsequent antigen-specific adaptive immune responses represent an additional defense that develops with time throughout the course of infection. The cellular-mediated facet of the adaptive branch of the immune system is dependent on the recognition of intracellularly-derived viral peptides by specific cytotoxic T lymphocytes (CTLs). Specifically, intracellularly processed viral peptides are presented on the cell surface in the context of major histocompatibility complex class I (MHC-I) molecules. This membrane bound MHC-I antigen complex can crosslink with receptors present on surveilling CTLs, which triggers the necessary anti-viral mechanisms necessary to resolve infection [3].

Many viruses have developed sophisticated methods to sabotage and evade the host cell’s anti-viral defenses, including the adaptive CTL response [4]. This is perhaps best exemplified by the identification of numerous viral genes that block MHC-I antigen presentation by reducing the expression of MHC-I components, limiting antigen loading onto MHC-I, or blocking intracellular transport of MHC-I to the cell surface. Moreover, viral proteins that promote MHC-I internalization and/or degradation have also been identified [4]. Collectively, these diverse strategies allow the infected cell to evade recognition and killing by CTLs, thereby promoting viral persistence.

Epstein–Barr virus (EBV) is a gamma-herpesvirus first identified from a case of Burkitt’s lymphoma [5]. It is a large virus with an enveloped capsid and a double-stranded DNA genome that infects B cells and mucosal epithelia to induce cellular proliferation. Notably, EBV infections are highly prevalent and the virus persists for the lifetime of the infected individual, primarily because of the extensive repertoire of immune evasion mechanisms deployed by this complex virus [6]. Furthermore, EBV is also a causative agent for multiple types of lymphomas, nasopharyngeal carcinomas, and EBV-associated gastric carcinomas (EBVaGCs). Worldwide, EBV infection is responsible for about 1.5% of all human cancers [7].

EBV-associated cancers consistently express a subset of viral genes, which primarily correspond to overlapping subsets of latency-associated factors [7]. Some of these products, including EBV nuclear antigen 1 (EBNA-1), are absolutely essential in maintaining persistence of the viral genome as the infected cell proliferates [8]. These viral proteins, in conjunction with small virally encoded RNAs, promote carcinogenesis by increasing cell proliferation, promoting cell motility, and inhibiting apoptosis [7]. Moreover, some of these viral genes also contribute to the evasion of adaptive immunity, suggesting a model in which these EBV-encoded products contribute to the ability of EBV-associated cancers to evade anti-tumor CTL responses [6,7,9].

An etiological role for EBV in gastric cancer (GC) was first identified in 1990 [10]. Since then, EBV has been implicated in causing approximately 9% of GCs worldwide [11]. Indeed, EBVaGCs are a distinct clinical and molecular subtype of GC, exhibiting male predominance, anatomical subsite preference to the cardia rather than antrum, extensive promoter hypermethylation, and improved prognosis compared to other subtypes of GC [11,12,13].

Expression of EBV proteins and microRNAs (miRNAs) in EBVaGCs are thought to contribute to gastric carcinogenesis [12,14]. EBVaGCs express low levels of EBNA-1, and frequently other latency-associated proteins, including latent membrane protein 1 and 2A (LMP-1 and LMP-2A, respectively) [12]. In addition, more recent studies have also detected consistent expression of mRNAs for a subset of EBV lytic genes in EBVaGCs [15,16]. Importantly, the most highly transcribed viral RNAs map within the BamHI-A region of the genome [17], including the BamHI-A rightward transcripts (BARTs) that encode for 44 intronic viral miRNAs [18]. Interestingly, a clear role of the EBV-encoded BART miRNAs for inhibiting anti-viral CD4+ and CD8+ T cell response during primary infection of B cells has been demonstrated [19,20]. Intriguingly, EBV-encoded miRNAs have also been implicated in reducing the expression and function of multiple components of the MHC-I antigen presentation apparatus in B cells, including the direct targeting of the mRNA encoding the transporter of antigenic peptide subunit 2 (TAP2) [19]. Whether these viral miRNAs contribute to immune evasion of EBV-associated cancers by impacting expression of the MHC-I antigen presentation apparatus has not been investigated directly.

In this study, we used data from The Cancer Genome Atlas (TCGA) gastric adenocarcinoma cohort to determine if the presence of EBV alters the steady state levels of mRNA for all the components of the MHC-I antigen presentation apparatus. The TCGA obtained fresh frozen tissues from nearly 400 GCs that were not treated with prior radiotherapy or chemotherapy. These samples, and in some cases adjacent non-malignant control tissue, were characterized by multiple genomics platforms, including mRNA sequencing, miRNA sequencing, whole exome sequencing, whole genome sequencing, array-based DNA methylation analysis, and array based somatic copy number analysis [13,17]. The results of this comprehensive molecular evaluation led to the classification of GCs into four subtypes: EBVaGC, chromosomal instability (CIN), genomically stable (GS), or microsatellite instability (MSI). EBV status was determined using mRNA, miRNA, and exome and whole-genome sequencing, and each of these platforms yielded concordant results. This dataset provides unique resources for studies that aim to understand the impact of EBV on GC.

Using the TCGA RNA-sequencing data, we determined the influence of EBV on the expression levels of the classical and non-classical human leukocyte antigen (HLA) heavy chain genes and the β2 microglobulin light chain (B2M) gene. Furthermore, we also assessed the impact of EBV on expression levels of genes encoding essential factors involved in the production, transport, and loading of antigenic peptides, including TAP1/2, tapasin, ERp57, calreticulin, calnexin, and endoplasmic reticulum aminopeptidases 1 and 2 (ERAP1/2). Our results showed that EBVaGCs generally exhibited higher levels of all MHC-I components compared to normal control tissues or other GC subtypes. Taken together, these data suggest that the EBV-encoded products expressed in EBVaGC do not effectively reduce the levels of mRNA encoding MHC-I and the loading apparatus in primary human gastric tumors. In addition, presentation of viral antigens by MHC-I may also contribute to the improved outcomes observed in EBVaGC in comparison to other GC subtypes.

## 2. Materials and Methods

### 2.1. RNA Expression Comparisons and Statistical Analysis

Level 3 RNA-Sequencing by Expectation Maximization (RSEM) normalized Illumina HiSeq RNA expression data and accompanying merged clinical data for the TCGA/PanCancer Atlas gastric carcinoma (STAD) cohort was downloaded from the Broad Genome Data Analysis Centers Firehose server (Cambridge, MA, USA; https://gdac.broadinstitute.org/) [21]. For all genes, the gene level Firehose dataset was used. Normalized mRNA expression data were extracted into Excel (Microsoft Corporation, Redmond WA, USA) and the GS subtypes were curated on the basis of the clinical data subtype characterization reported for the TCGA PanCancer Atlas 2018 dataset of 440 STAD samples, as downloaded from cBioPortal (http://www.cbioportal.org/).

For each gene analyzed, primary patient samples were subdivided into five groups on the basis of GC subtype (EBV-associated gastric adenocarcinoma (EBVaGC), chromosomal instability (CIN), genomically stable (GS), or microsatellite instability (MSI)) or classification as normal control gastric tissue. Patient samples with unknown EBV status were omitted from our calculations. This resulted in 30 EBVaGC, 223 CIN, 50 GS, 73 MSI, and 35 normal control samples with data available for gene expression analysis.

Expression levels of selected mRNAs were compared across each gastric cancer subtype and normal control tissue using Mann–Whitney U tests in RStudio (Version 1.2.1335; RStudio Inc., Boston, MA, USA), and derived *p*-values were corrected for multiple testing using the Benjamini–Hochberg method. The threshold of significance for reported FDR (false discovery rate) values was set at 0.1. Boxplots and heatmaps were generated using the ggplot2 package [22] in RStudio. Final figure layouts were performed with CorelDRAW (Corel Corporation, Ottawa, ON, Canada).

For overall analysis of the effect of patient EBV status, sex, and age on mRNA expression of selected MHC-I genes, high and low expression groups across the entire TCGA GC cohort were defined on the basis of division by the 50th percentile. The difference between high and low expression groups for EBV status and patient sex was compared using Pearson’s chi-squared test with Yates’ continuity correction. Age was compared between high and low gene expression groups using a Mann–Whitney–Wilcoxon test, with a *p*-value significance threshold of 0.05. Final figure layout was performed using Excel.

### 2.2. Correlation Matrix

Level 3 RSEM normalized RNA-sequencing (RNA-seq) data for the genes listed above were extracted from the TCGA database and processed for the EBVaGC, CIN, GS, and MSI samples as detailed above. Normalized EBV RNA expression data for 26 samples was downloaded from Pandya et al. [23]. Pairwise Spearman correlation was performed for each of the indicated genes involved in the MHC-I-dependent antigen presentation system or its regulation. Correlations were performed using RStudio (version 1.2.1335) utilizing the ggplot2 package [22]. The correlation matrix figure was assembled into final form using Adobe Illustrator (Adobe Systems Inc., San Jose, CA, USA).

## 3. Results

### 3.1. Correlation between mRNA Expression Levels of MHC-I Genes, EBV Status, and Clinical Variables in Human Gastric Cancers

Classical MHC-I heterodimers are comprised of a heavy chain encoded by one of three genes (HLA-A, -B, and -C) and the β2 microglobulin (B2M) encoded invariant light chain [4]. Antigen loading requires many factors, including the peptide transporter complex comprised of TAP1 and TAP2 and the bridging factor tapasin (TAPBP) [4]. In various systems, EBV has been shown to reduce the steady state level of one or more mRNAs encoding heavy chain [24,25], as well as TAP1 and/or TAP2 [19,24]. As a first step in determining the impact of EBV status on MHC-I gene expression, we first analyzed the Illumina HiSeq RNA expression data from the TCGA STAD cohort for expression of HLA-A, -B, -C, B2M, and the TAP genes (Table 1). Patients were divided into high and low expression groups across the entire cohort on the basis of the 50th percentile of expression for each gene. Unexpectedly, EBVaGC samples were significantly overrepresented in the high expression group for each of these seven MHC-I pathway genes compared to EBV negative GCs (*p*< 0.05).

Because EBVaGC is more prevalent in males and often occurs in younger patients [26], we wanted to exclude a potential bias in expression due to the influence of either sex or age. We similarly analyzed the relationship between those clinical variables and expression of these seven MHC-I pathway genes (Table 1). Our results indicated that sex and age were not significantly associated with mRNA expression levels (*p* > 0.05). Thus, the higher levels of mRNA for these MHC-I pathway genes do not appear to be related to skewing based on the clinical characteristics of the EBVaGC subset of patients.

### 3.2. Impact of EBV Status on MHC-I Heavy Chain mRNA Expression in Human Gastric Cancers

**Figure 1:**
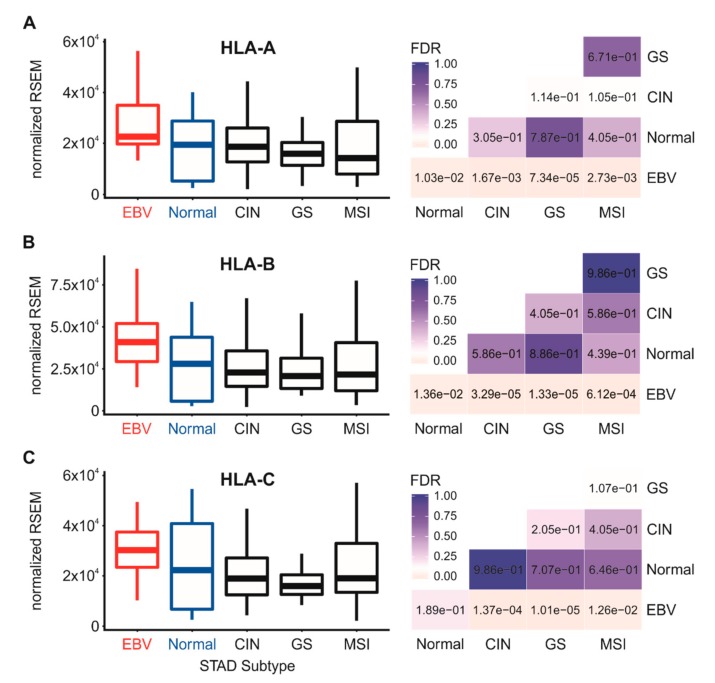
Expression of classical MHC-I heavy chain gene mRNA in gastric carcinoma subtypes and normal gastric tissue. RNA-Sequencing by Expectation Maximization (RSEM) normalized data for the HLA-A (**A**), HLA-B (**B**) and HLA-C (**C**) MHC-I heavy chain genes were extracted from The Cancer Genome Atlas (TCGA) database for the TCGA/PanCancer Atlas gastric/stomach adenocarcinoma (STAD) cohort for EBV-associated gastric carcinomas (EBVaGCs), normal control tissues, and three other gastric cancer (GC) subtypes. False discovery rate (FDR)-adjusted *p*-values for each statistical comparison are shown on the right for each gene panel. CIN: chromosomal instability; GS: genomically stable; MSI: microsatellite instability.

**Figure 2:**
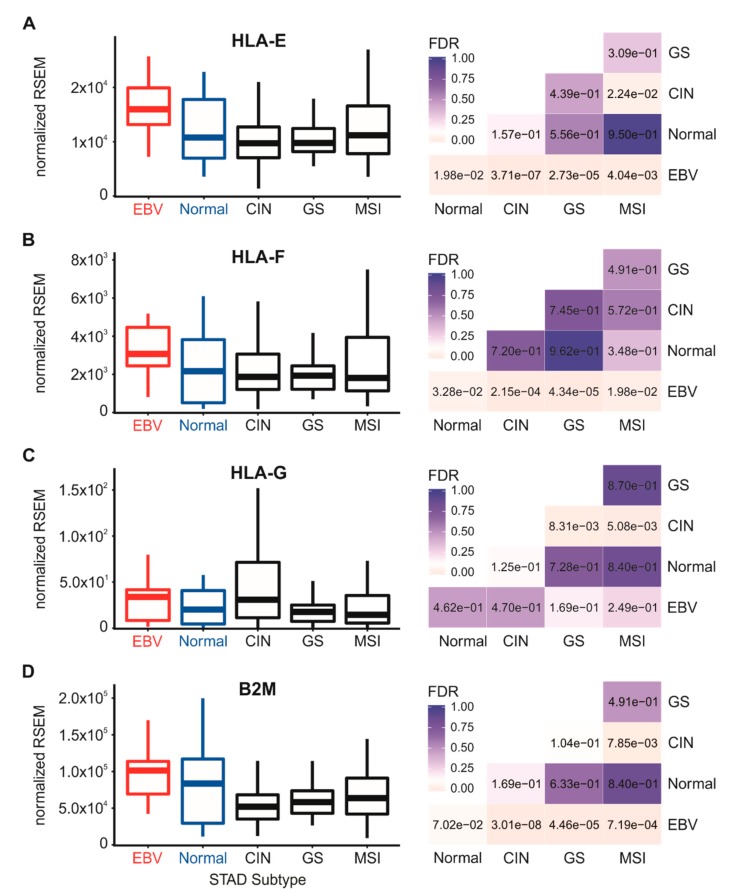
Expression of non-classical MHC-I heavy chain genes and light chain in gastric carcinoma subtypes and normal gastric tissue. Normalized RNA-seq data for the HLA-E (**A**), HLA-F (**B**) and HLA-G (**C**) MHC-I heavy chain and B2M (**D**) light chain genes were extracted from the TCGA database for the STAD cohort for EBVaGCs, normal control tissues, and three other GC subtypes. FDR-adjusted *p*-values for each statistical comparison are shown on the right for each gene panel.

We next analyzed the Illumina HiSeq RNA expression data for expression of the three classical heavy chain genes, HLA-A, -B, and -C, across the four TCGA-defined GC subsets and normal control tissue (Figure 1). EBVaGC samples expressed significantly elevated or at least comparable levels of HLA-A, -B, and -C mRNA compared to normal control tissues or other GC subtypes. Similarly, higher or comparable levels of mRNA expression of the non-classical heavy chain genes, HLA-E and HLA-F, were observed in EBVaGC samples with respect to normal control tissues and other GC subtypes (Figure 2). This agrees with a previous report that HLA-A mRNA levels are increased in EBVaGC [27] and another report that HLA-E mRNA levels are increased [25]. In contrast to the other heavy chains, no significant difference in the mRNA levels of HLA-G was apparent between EBVaGC, normal control tissues, or other GC subtypes. However, the relative normalized mRNA expression level of this gene was 100- to 1000-fold lower than the other heavy chain genes, suggesting that its contribution to antigen presentation is minimal in the context of gastric epithelia (Figure 2). Collectively, these results indicate that not only is the presence of EBV in GCs not correlated with a reduction of steady state mRNA from the MHC-I loci, it is often correlated with increased expression.

### 3.3. Impact of EBV Status on the Expression of mRNA Encoding Other Components of the MHC-I Antigen Presentation Apparatus in Human Gastric Cancers

**Figure 3:**
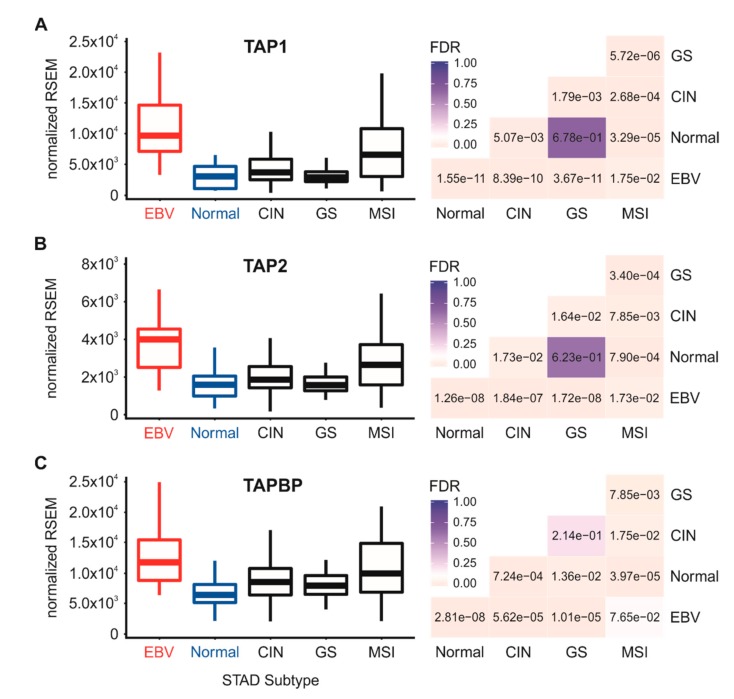
Expression levels of the TAP genes involved in MHC-I-dependent antigen presentation in gastric carcinoma subtypes and normal gastric tissue. Normalized RNA-seq data for the TAP1 (**A**), TAP2 (**B**) and TAPBP (**C**) genes involved in MHC-I-dependent antigen presentation were extracted from the TCGA database for the STAD cohort for EBVaGCs, normal control tissues, and three other GC subtypes. FDR-adjusted *p*-values for each statistical comparison are shown on the right for each gene panel.

**Figure 4:**
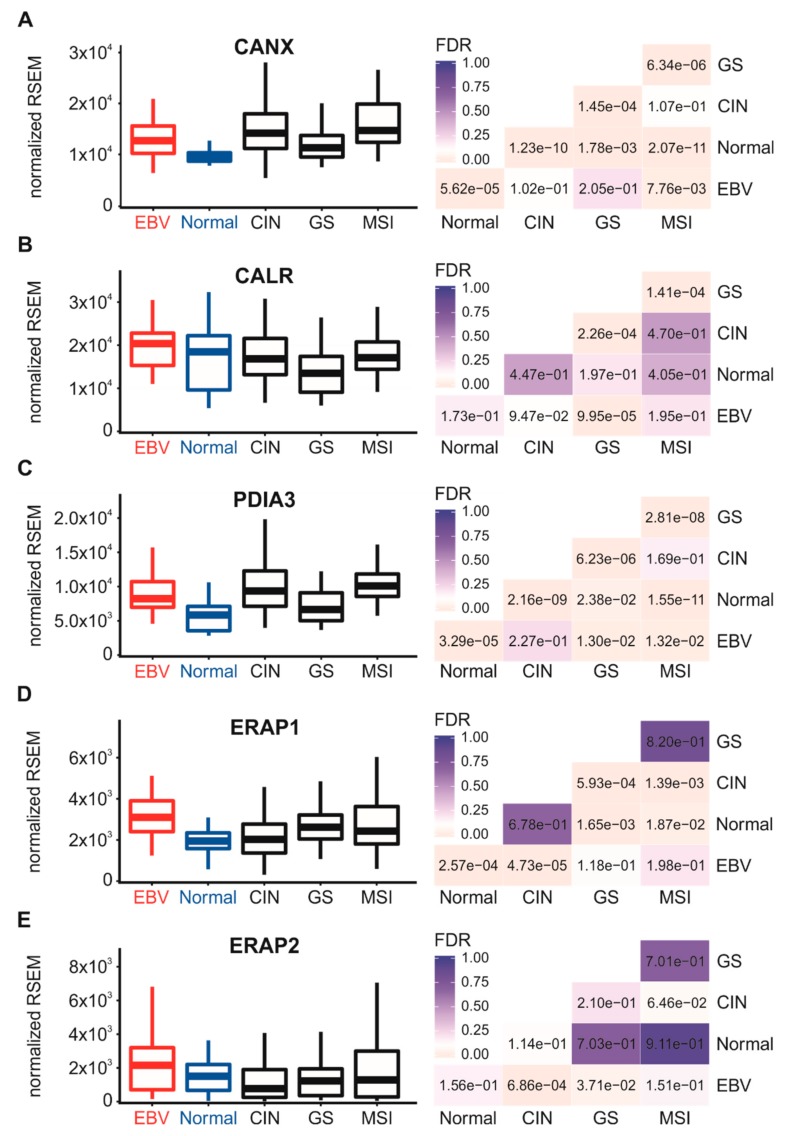
Expression levels of other genes involved in MHC-I-dependent antigen loading in gastric carcinoma subtypes and normal gastric tissue. Normalized RNA-seq data for the CANX (**A**), CALR (**B**), PDIA3 (**C**), ERAP1 (**D**) and ERAP2 (**E**) genes involved in MHC-I-dependent antigen presentation were extracted from the TCGA database for the STAD cohort for EBVaGCs, normal control tissues, and three other GC subtypes. FDR-adjusted *p*-values for each statistical comparison are shown on the right for each gene panel.

The process of MHC-I heavy chain folding and dimerization with the invariant β2 microglobulin light chain occurs within the endoplasmic reticulum through a process that is dependent on binding to an antigenic peptide [4]. The MHC-I peptide-loading complex consists of the MHC-I heterodimer; the peptide transporter complex comprised of TAP1 and TAP2; the bridging factor tapasin (TAPBP); the endoplasmic reticulum aminopeptidases (ERAP1 and 2); and the chaperones calreticulin (CALR), calnexin (CANX), and ERp57 (PDIA3). EBV-encoded miRNAs have been reported to downregulate TAP1, TAP2, and ERAP2 mRNA in infected primary B cells [19], and the TAP2 mRNA was similarly reduced in EBV-associated nasopharyngeal carcinomas [24]. However, less is known about the effect of EBV status on the expression of the other components necessary for MHC-I antigen loading and presentation. Analysis of the TCGA STAD cohort data revealed high levels of transcripts for the B2M gene encoding β2 microglobulin (Figure 2D), TAP1, TAP2, TAPBP (Figure 3), and the genes encoding ERAP1/2, calreticulin, calnexin, and ERp57 in EBVaGC samples (Figure 4).

All genes were expressed at higher or comparable mRNA levels in EBVaGC samples with respect to normal control tissues or other GC subtypes. Thus, mRNA levels for all components of the MHC-I loading complex are present in EBVaGCs at levels that are at least as high or significantly higher than normal control tissues. These results contrast with studies that have reported EBV miRNA-dependent decreases in TAP1, TAP2, and ERAP2 mRNA levels during infection of primary B cells [19,20].

### 3.4. Higher Levels of Lymphocytes, and Interferon γ are Present in EBV-Associated Gastric Carcinomas

Interferon γ (IFN-γ), the product of the IFNG locus, can coordinately induce transcription of many of the genes involved in MHC-I-dependent antigen processing and presentation, providing enhanced immune surveillance under inflammatory conditions [28]. Given our observation that mRNA levels of all MHC-I loading and presentation genes are expressed at similar or significantly higher levels in EBVaGCs compared to normal control tissues, we hypothesized that infiltrating lymphocytes producing IFN-γ could be present at higher levels in EBVaGCs compared to the other GC subtypes or normal control tissues.

**Figure 5:**
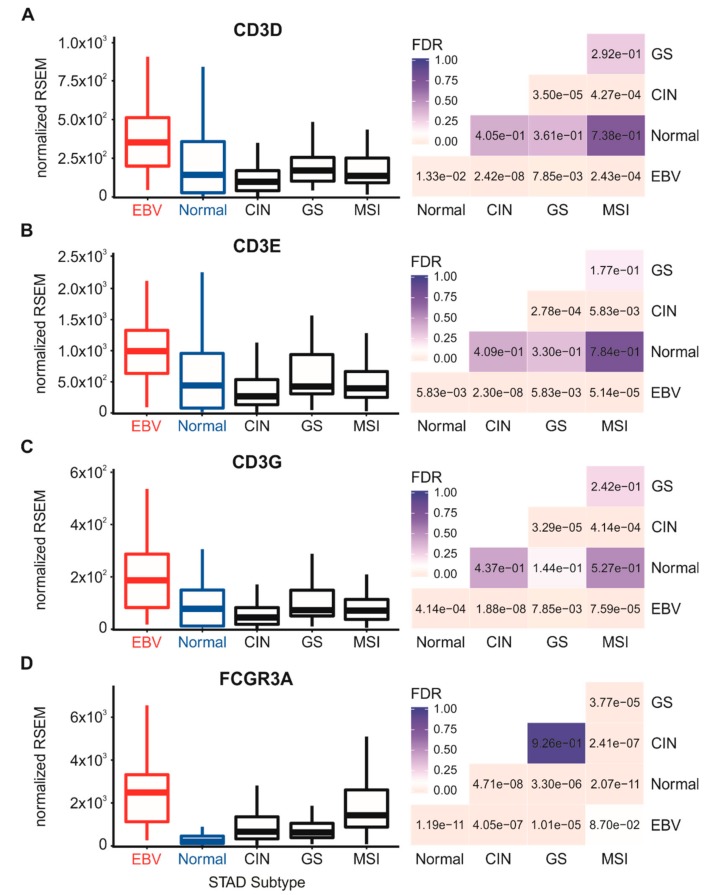
Detection of tumor infiltrating T cells and natural killer (NK) cells in gastric carcinoma subtypes and normal gastric tissue. Normalized RNA-seq data for genes indicative of tumor infiltrating T cells including CD3D (**A**), CD3E (**B**), and CD3G (**C**), or FCGR3A (**D**) for NK cells were extracted from the TCGA database for the STAD cohort for EBVaGCs, normal control tissues, and three other GC subtypes. FDR-adjusted *p*-values for each statistical comparison are shown on the right for each gene panel.

We first assessed the relative proportion of T and natural killer (NK) cells in each sample, which are the primary producers of IFN-γ. Similarly to what has been performed by others [29], we analyzed the expression levels of the mRNA encoding CD3 (CD3D, CD3E, and CD3G) and CD16a (FCGR3A) as surrogate markers for T and NK cells, respectively (Figure 5). EBVaGC samples showed significantly increased mRNA expression from all three genes encoding subunits of CD3 versus normal control or other GC subtype samples. For the NK cell marker CD16a, relative normalized expression of FCGR3A mRNA was very high and significantly greater in EBVaGC cells versus normal control tissues or the other GC subtypes. However, the relative normalized mRNA levels for these lymphocyte-specific mRNAs were 10–100 fold lower than the classical MHC-I heavy chain and light chain mRNAs (cf. Figure 1 and Figure 2 with Figure 5), suggesting that infiltrating lymphocytes comprised only a fraction of the cells in the tumor samples. Nevertheless, these results suggest that there was greater infiltration of T and NK cells in the tumor microenvironments of EBVaGCs compared to normal gastric tissue or the other GC subtypes, which could be responsible for stimulating tumor cells to upregulate essential components of the MHC-I presentation system.

**Figure 6:**
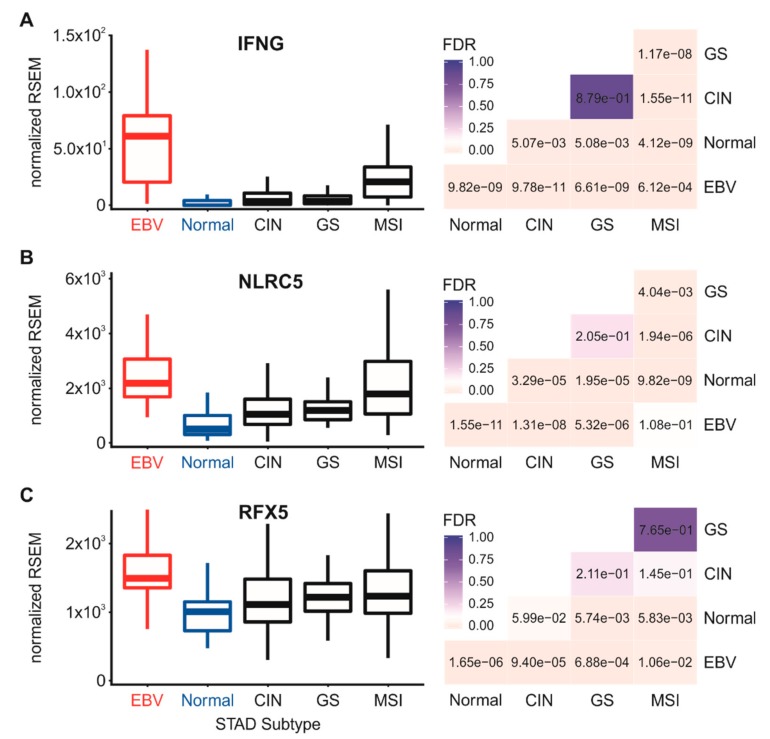
Expression of mRNA encoding IFN-γ and transcription factors involved in regulating the interferon-dependent activation of MHC-I-dependent antigen presentation and loading genes. Normalized RNA-seq data for the IFN-γ (IFNG) gene (**A**) and the genes encoding the nucleotide-binding oligomerization domain (NOD)-like receptor caspase recruitment domain containing protein 5 (NLRC5)/MHC-I transactivator (CITA; panel (**B**)) and Regulatory Factor X5 (RFX5; panel (**C**)) transcription factors involved in interferon-induced activation of expression of genes involved in MHC-I-dependent antigen presentation were extracted from the TCGA database for the STAD cohort for EBVaGCs, normal control tissues, and three other GC subtypes. FDR-adjusted *p*-values for each statistical comparison are shown on the right for each gene panel.

We next looked at levels of IFN-γ mRNA to determine if these lymphocytes were producing this proinflammatory cytokine (Figure 6A). Although expression of IFN-γ mRNA in these samples was low on the basis of the normalized relative mRNA reads, it was significantly higher in EBVaGC samples versus normal control tissues or the other GC subtypes (Figure 6A), which confirms a previous study [30]. Taken together, these results indicate that EBVaGCs have a significantly increased level of lymphocytes infiltrating into their respective tumor microenvironments that likely produce an increased level of IFN-γ. Thus, the observed upregulated expression of mRNA encoding essential MHC-I loading and presentation components in EBVaGC may be a consequence of exposure of the carcinoma cells to intratumoral IFN-γ.

### 3.5. Impact of EBV Status on mRNA Levels of the Transcriptional Regulators of MHC-I Gene Expression

Transcriptional control of MHC-I genes in epithelial cells is dependent on the master transcriptional regulator nucleotide-binding oligomerization domain (NOD)-like receptor caspase recruitment domain containing protein 5 (NLRC5)/MHC-I transactivator (CITA). Beyond its role in the constitutive expression of MHC-I, NLRC5 is rapidly induced by IFN-γ and essential for IFN-γ induction of MHC-I transcription [31]. In agreement with the high levels of MHC-I and related genes, analysis of the TCGA data revealed significantly higher levels of NLRC5 in EBVaGC compared to normal control samples or other GC subtypes (Figure 6B). In addition, Regulatory Factor X5 (RFX5)—another key transcriptional regulator of MHC-I genes [32]—was similarly expressed at significantly higher levels in EBVaGC samples with respect to normal control tissues or other GC subtypes (Figure 6C). Thus, the upregulated mRNA levels of NLRC5, RFX5, and subsequent increases in mRNA levels of the MHC-I antigen presentation genes and related genes required for antigen loading and presentation observed in EBVaGCs could be consequences of increased IFN-γ exposure in an inflamed tumor microenvironment.

**Figure 7:**
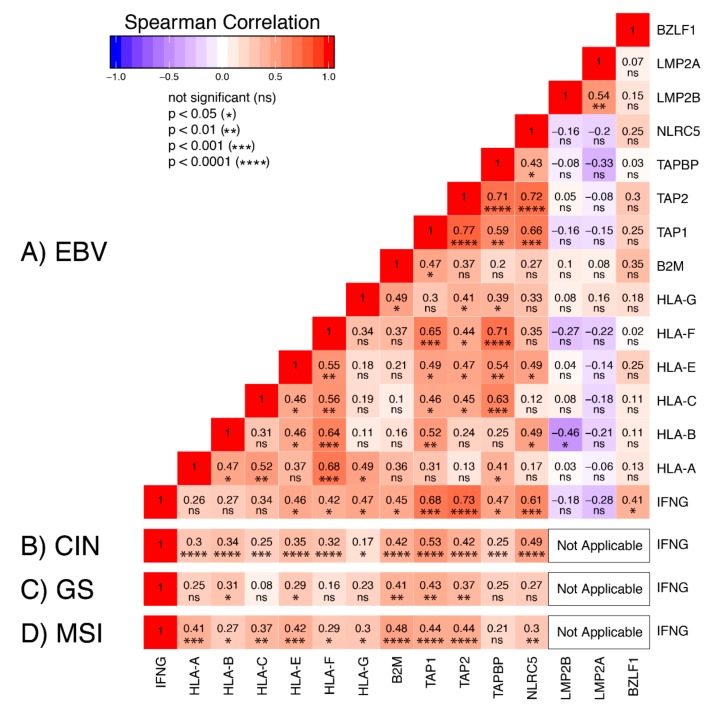
Correlation matrix of selected genes involved in the MHC-I antigen presentation pathway. Heatmap of Spearman correlation analysis of mRNA expression of the indicated MHC-I pathway genes in EBVaGC (**A**). Comparisons with EBV genes reported to antagonize interferon-γ response are also shown. For comparison, Spearman correlations between mRNA levels for interferon-γ (IFNG) and MHC-I pathway genes are also shown for the CIN (**B**), GS (**C**), and MSI (**D**) subtypes. RSEM normalized RNA-seq data for the genes listed above were extracted from the TCGA database for the STAD cohort for EBVaGCs. Pairwise spearman correlations were performed. Numbers in boxes indicate Spearman’s rank correlation coefficient of analyzed gene pairs and *p*-values.

To investigate the relationship between IFN-γ and mRNA expression of MHC-I-related genes, we generated a correlation matrix for the EBVaGC samples (Figure 7A). As expected [31], IFNG mRNA expression was highly correlated with NLRC5 mRNA levels across the EBVaGC samples. Expression of the mRNAs encoding the non-classical MHC-I heavy chains (HLA-E, -F, and -G), the light chain (B2M), and peptide transporter complex components (TAP1, 2, and TAPBP) were also significantly correlated in a pairwise fashion with IFNG mRNA levels. Unexpectedly, expression of mRNA encoding the classical MHC-I heavy chains (HLA-A, -B, and -C) was not significantly correlated with IFNG in the EBVaGCs (Figure 7A). In comparison, mRNA levels of all MHC-I pathway genes were correlated with IFNG in the CIN GCs (Figure 7B), and all but TAPBP mRNA levels were correlated in the MSI GCs (Figure 7D). Fewer correlations were present in the GS GCs, which could reflect the low levels of IFNG mRNA in those samples (Figure 7C). Despite the fact that the correlation matrix illustrated a high degree of coordination of the transcription of the MHC-I apparatus in GCs in general, the absence of the expected correlation between IFNG mRNA and HLA-A, -B, and -C mRNA levels in the EBVaGC samples suggests that the presence of EBV could exert some modest antagonism on their expression.

Interestingly, the EBV encoded LMP2A, LMP2B, and BamHI-Z region leftward open reading frame 1 (BZLF1) products are known antagonists of the IFN-γ response pathway [33,34] and they are expressed at variable levels in EBVaGC [15,23]. We expanded our pairwise comparisons to determine if mRNA levels of any of these viral genes were correlated with a reduction in the IFN-γ response (Figure 7A). We detected a significant inverse correlation between LMP2B and HLA-B, but not between the EBV mRNAs and any other MHC-I pathway genes (Figure 7A). Thus, despite relatively high mRNA expression levels of these EBV-encoded transcripts in many of the EBVaGCs [15,23], only LMP2B expression was correlated with a reduction of MHC-I mRNA levels, and this was restricted to only HLA-B.

## 4. Discussion

EBV-associated cancers express viral genes associated with latency, but also may express additional viral genes involved in the lytic cycle of infection. Functionally, these genes are suspected to have profound effects on cellular gene expression and cell regulation, contributing to the altered cell growth, survival, metabolism, and other abnormal characteristics of cancerous cells. Moreover, viral proteins could also serve as foreign antigenic peptides, leading to a more effective anti-cancer adaptive immune response. Alternatively, viral effectors could reduce immunogenicity by blocking antigenic peptide presentation, thereby enhancing the evasion of anti-tumor immune responses.

MHC-I-dependent presentation of viral antigens or tumor-derived neo-antigens on the cell surface is a key component of immune surveillance for both infection and cancer. Stable surface expression of MHC-I requires loading of heavy chain/light chain heterodimers with a peptide in the endoplasmic reticulum. The loading complex consists of multiple essential factors, including the peptide transporter TAP; the bridging factor tapasin; and the chaperones calreticulin, calnexin, and ERp57. Mutations or reduced expression of any of these components can compromise MHC-I-dependent antigen presentation, allowing the infected or cancerous cell to escape the CTL response [4]. During lytic infection, EBV encodes many different proteins that negatively impact MHC-I-dependent antigen presentation in a variety of interesting ways [6]. Collectively, these contribute to the ability of this very successful human pathogen to infect over 90% of the adult population and maintain infection over the lifetime of the infected individual [35].

In the context of EBV-associated human epithelial cancers, the majority of viral lytic genes known to affect MHC-I expression and function are not expressed, or are expressed at extremely low levels [15,36]. However, virally encoded miRNAs are highly expressed in EBV-dependent cancers, representing a large fraction of total miRNAs present in the cell [37]. Furthermore, these viral miRNAs are known to contribute to EBV-mediated evasion of the adaptive immune response in other contexts [19,20]. Mechanistically, part of this process occurs through the downregulated expression of the MHC-I genes or other components necessary for antigen loading and/or presentation, and this has been reported in both EBVaGCs [25] and EBV-associated nasopharyngeal cancers [24]. Thus, miRNA-mediated downregulation of the MHC-I apparatus could contribute to the ability of EBV-associated human cancers to evade anti-tumor CTL responses.

Although cell culture models allow for detailed studies on the downregulation of essential components involved in the MHC-I antigen presentation system, how closely these effects mirror what is observed in actual EBV-associated gastric tumors is not known. This could be further impacted by the fact that many of the virally encoded BART miRNAs are expressed at significantly increased levels in the context of actual tumors as compared to tissue culture models, which could greatly impact the extent and breadth of their functions [38,39]. Furthermore, the lack of tumor microenvironment and associated heterogeneity in these in vitro models could also limit accurate assessment of these complex processes, which are heavily influenced by extracellular stimuli. Therefore, in this study, our goal was to determine if primary EBVaGCs exhibited a reduction in steady state levels of mRNA encoding MHC-I components, as observed in other contexts of EBV infection.

Using data from nearly 400 primary human gastric tumors, we provided evidence that EBVaGCs display high mRNA levels for MHC-I components, including heavy and light chains, as well as factors required for loading. Indeed, EBVaGCs exhibited increases, rather than decreases in the mRNA levels for virtually all MHC-I components as compared to normal control tissues or other GC subtypes (Figure 1, Figure 2, Figure 3 and Figure 4). Existing data on the effects of EBV status on mRNA expression levels is limited. One study reported an increase in HLA-A mRNA in EBVaGC [27]. Another reported that mRNA levels for HLA-A and -B, but not -C, were reduced in EBVaGCs compared to other GCs, whereas HLA-E was upregulated [25]. That study used a smaller cohort and reverse transcriptase PCR, rather than RNA-seq to detect changes in mRNA levels, which may contribute to the differences with our observations. That study also did not look for changes in any of the other MHC-I components that we investigated here. Interestingly, contradictory effects of EBV on expression of the MHC-I apparatus in nasopharyngeal carcinomas have similarly been reported [24,40,41]. Given the systematic upregulation of mRNA levels encoding MHC-I components, our results contradict existing paradigms of virally mediated immune evasion and suggest that in the context of an actual in vivo human tumor setting, the presence of EBV results in increased expression of mRNA encoding MHC-I-dependent antigen presentation components in GCs.

The enhanced expression levels of mRNA encoding MHC-I and components of the antigen loading complex could be via the activation of the Janus kinase-signal transducer, activator of transcription ((JAK/STAT) and interferon regulatory factor 1 (IRF-1) signal transduction pathway through exposure to IFN-γ [28]. Intratumoral IFN-γ originates from tumor-infiltrating lymphocytes. Using surrogate markers, we also found evidence for higher levels of T cells and NK cells in EBVaGCs versus other GC subtypes (Figure 5), which agrees with reported histological characteristics [30,42,43]. Our analysis also revealed low but detectable levels of mRNA for IFN-γ in EBVaGC samples, which were nevertheless significantly higher than normal control tissues or other GC subtypes (Figure 6).

Increased levels of intratumoral IFN-γ is also consistent with the observed increased expression of NLRC5 and RFX5 mRNAs, which encode two key global regulators of MHC-I pathway genes that are themselves upregulated in epithelial cells in response to IFN-γ (Figure 6). In addition, a strong correlation was identified between IFNG mRNA level and mRNA levels for many of the genes involved in MHC-I presentation or regulation (Figure 7). This observation was rather surprising, as EBV encodes multiple antagonists of the IFN-γ response pathway. Specifically, the EBV immediate early protein BZLF1 is known to abrogate the IFN-γ response, inhibiting IFN-γ induction of transcription [33]. Similarly, the EBV latency proteins LMP2A and LMP2B both inhibit the IFN-γ response by increasing turnover of the IFN-γ receptor, also inhibiting the transcriptional response to IFN-γ in epithelial cells [34]. Nevertheless, the elevated levels of MHC-I components expressed in EBVaGCs suggest that none of these EBV proteins block the IFN-γ induction of MHC-I genes in these human tumors. Although mRNA expression of LMP2A, LMP2B, and BZLF1 was detected in many of the EBVaGC samples, their levels were not inversely correlated with the expression level of any of the MHC-I genes across the individual samples, with the sole exception of LMP2B and HLA-B (Figure 7A). Thus, these viral genes may simply be expressed at levels below the threshold needed to effectively block this signal transduction pathway. Interestingly, EBVaGCs were recently reported as exhibiting the highest IFN-γ gene response signature of all the GC subtypes, supporting the conclusion that EBV products do not cripple the IFN-γ response in these cancers [13,44]. Several other studies of cell lines derived from EBVaGC have similarly demonstrated evidence of an intact IFN-γ gene response, despite the presence of EBV-encoded products [45,46].

As mentioned above, EBV expresses a plethora of viral miRNAs. Indeed, the BART miRNAs account for >10% of the total pool of miRNAs in EBV-positive epithelial tumor cells [37], suggesting that they extensively alter the miRNAome and contribute to carcinogenesis. The majority of EBVaGCs express high levels of nearly all the BART miRNAs [23], including the miRNA-BART17 that was previously shown to directly target TAP2 mRNA in infected primary B cells [19]. The BART17 miRNA was also reported as being one of the most highly expressed of BART-encoded miRNAs and was present in all 17 EBVaGCs tested [47]. Nevertheless, TAP2 mRNA expression remains highly upregulated in EBVaGC compared to normal control tissues or other GC subtypes (Figure 3). Thus, in vitro studies may not reflect the reality of actual tumors, particularly in cases where the tumor milieu contains a heterogeneous mixture of cells, specifically immune cells that infiltrate the tumor microenvironment and produce potent inflammatory mediators such as IFN-γ that have the ability to upregulate the transcription of proposed mRNA targets.

It is important to note that the presence of high levels of mRNA for all components of the MHC-I antigen loading and presentation apparatus does not necessitate a high level of protein expression, correct protein localization, or actual protein function in EBVaGCs. Indeed, EBV encodes several additional proteins that interfere with MHC-I-dependent antigen presentation at the protein level. These include inhibition of TAP function by BNLF2a [48], downregulation of cell surface MHC-I for certain haplotypes by reducing transport from the endoplasmic reticulum by BILF1 [49], and BDLF3-induced ubiquitination of MHC molecules and their subsequent degradation by the proteasome [50].

Interestingly, although BDLF3 expression is rarely detected in EBVaGCs, many EBVaGCs tested expressed significant levels of mRNA for both BNLF2a and BILF1 [15,44,51], and BNLF2a protein was expressed in two of three EBVaGC cell lines tested at levels comparable to those observed in a Burkitt’s lymphoma cell line induced to undergo EBV reactivation [51]. Although immunohistochemical analyses of MHC-I heavy and light chain expression of EBVaGCs are sparse, no significant differences in either classical heavy or light chain expression by immunohistochemistry were apparent in a study comparing 20 EBVaGCs with 28 EBV-negative GCs [52]. More recently, MHC-I heavy and light chain proteins were detected in the majority of EBVaGCs, whereas the majority of other GC types did not express detectable levels of the MHC-I proteins [53]. As deficiencies in peptide loading impair MHC-I transport to the cell surface [54], TAP function is also not likely to be impacted. Thus, it seems likely that the level of expression of these viral antagonists of MHC-I-dependent antigen presentation are not sufficient in being able to grossly impact the MHC-I presentation system in EBVaGCs.

In conclusion, MHC-I-dependent antigen presentation is critical for CD8+ T cell responses, which are essential for the control and clearance of virally infected or cancerous cells. The presence of non-self-derived viral antigens, combined with intact expression of the MHC-I antigen presentation complex and increased levels of infiltrating T cells, may contribute to the observation that patient outcomes are markedly better for EBVaGCs versus other subtypes of GCs. This could be quite analogous to human papillomavirus-dependent oropharyngeal cancers, which show many similar immunological features and improved patient survival compared to their virus-negative counterparts [55,56].

## Figures and Tables

**Table 1:** Analysis of correlations between mRNA levels of the indicated major histocompatibility complex class I (MHC-I) genes and patient characteristics, including Epstein–Barr virus (EBV) status, sex, and age. Green shading indicates statistical significance (*p* < 0.05).

Gene		Variables				
		EBV Status		Sex		Age
		Negative	Positive	Female	Male	Median (Max-Min)
***HLA-A***	High	164	24	63	128	68 (90–41)
	Low	186	6	68	124	66 (90–30)
	*p*-value	1.16 × 10^−3^		6.94 × 10^−1^		8.35 × 10^−2^
***HLA-B***	High	166	25	65	126	67 (90–35)
	Low	187	5	66	126	67 (90–35)
	*p*-value	2.85 × 10^−4^		1.00		6.71 × 10^−1^
***HLA-C***	High	165	26	66	125	68 (90–39)
	Low	188	4	65	127	66 (87–30)
	*p*-value	6.11 × 10^−5^		9.71 × 10^−1^		2.42 × 10^−1^
***B2M***	High	165	26	69	122	68 (90–39)
	Low	188	4	62	130	67 (90–30)
	*p*-value	6.11 × 10^−5^		4.95 × 10^−1^		7.52 × 10^−1^
***TAP1***	High	162	29	62	129	68 (90–35)
	Low	191	1	69	123	67 (90–30)
	*p*-value	2.61 × 10^−7^		5.42 × 10^−1^		2.20 × 10^−1^
***TAP2***	High	165	26	61	130	68 (90–39)
	Low	188	4	70	122	67 (90–30)
	*p*-value	6.11 × 10^−5^		4.50 × 10^−1^		4.69 × 10^−1^
***TAPBP***	High	169	22	64	127	68 (90–30)
	Low	184	8	67	125	67 (90–34)
	*p*-value	1.29 × 10^−2^		8.58 × 10^−1^		2.40 × 10^−1^

## References

[1] Yan N., Chen Z.J. (2012). Intrinsic antiviral immunity. Nat. Immunol..

[2] Takeuchi O., Akira S. (2009). Innate immunity to virus infection. Immunol. Rev..

[3] Tscharke D.C., Croft N.P., Doherty P.C., La Gruta N.L. (2015). Sizing up the key determinants of the CD8(+) T cell response. Nat. Rev. Immunol..

[4] Hansen T.H., Bouvier M. (2009). MHC class I antigen presentation: Learning from viral evasion strategies. Nat. Rev. Immunol..

[5] Epstein M.A., Achong B.G., Barr Y.M. (1964). Virus Particles in Cultured Lymphoblasts from Burkitt’s Lymphoma. Lancet.

[6] Ressing M.E., van Gent M., Gram A.M., Hooykaas M.J., Piersma S.J., Wiertz E.J. (2015). Immune Evasion by Epstein-Barr Virus. Curr. Top. Microbiol. Immunol..

[7] Farrell P.J. (2019). Epstein-Barr Virus and Cancer. Annu. Rev. Pathol..

[8] Frappier L. (2015). Ebna1. Curr. Top. Microbiol. Immunol..

[9] Zuo L., Yue W., Du S., Xin S., Zhang J., Liu L., Li G., Lu J. (2017). An update: Epstein-Barr virus and immune evasion via microRNA regulation. Virol. Sin..

[10] Burke A.P., Yen T.S., Shekitka K.M., Sobin L.H. (1990). Lymphoepithelial carcinoma of the stomach with Epstein-Barr virus demonstrated by polymerase chain reaction. Mod. Pathol..

[11] Murphy G., Pfeiffer R., Camargo M.C., Rabkin C.S. (2009). Meta-analysis shows that prevalence of Epstein-Barr virus-positive gastric cancer differs based on sex and anatomic location. Gastroenterology.

[12] Shinozaki-Ushiku A., Kunita A., Fukayama M. (2015). Update on Epstein-Barr virus and gastric cancer (review). Int. J. Oncol..

[13] Liu Y., Sethi N.S., Hinoue T., Schneider B.G., Cherniack A.D., Sanchez-Vega F., Seoane J.A., Farshidfar F., Bowlby R., Islam M. (2018). Comparative Molecular Analysis of Gastrointestinal Adenocarcinomas. Cancer Cell.

[14] Zhang J., Huang T., Zhou Y., Cheng A.S.L., Yu J., To K.F., Kang W. (2018). The oncogenic role of Epstein-Barr virus-encoded microRNAs in Epstein-Barr virus-associated gastric carcinoma. J. Cell Mol. Med..

[15] Borozan I., Zapatka M., Frappier L., Ferretti V. (2018). Analysis of Epstein-Barr Virus Genomes and Expression Profiles in Gastric Adenocarcinoma. J. Virol..

[16] Tang W., Morgan D.R., Meyers M.O., Dominguez R.L., Martinez E., Kakudo K., Kuan P.F., Banet N., Muallem H., Woodward K. (2012). Epstein-barr virus infected gastric adenocarcinoma expresses latent and lytic viral transcripts and has a distinct human gene expression profile. Infect. Agent Cancer.

[17] Cancer Genome Atlas Research Network (2014). Comprehensive molecular characterization of gastric adenocarcinoma. Nature.

[18] Skalsky R.L., Cullen B.R. (2015). EBV Noncoding RNAs. Curr. Top. Microbiol. Immunol..

[19] Albanese M., Tagawa T., Bouvet M., Maliqi L., Lutter D., Hoser J., Hastreiter M., Hayes M., Sugden B., Martin L. (2016). Epstein-Barr virus microRNAs reduce immune surveillance by virus-specific CD8+ T cells. Proc. Natl. Acad. Sci. USA.

[20] Tagawa T., Albanese M., Bouvet M., Moosmann A., Mautner J., Heissmeyer V., Zielinski C., Lutter D., Hoser J., Hastreiter M. (2016). Epstein-Barr viral miRNAs inhibit antiviral CD4+ T cell responses targeting IL-12 and peptide processing. J. Exp. Med..

[21] Broad Institute (2016). TCGA Genome Data Analysis Center Analysis: Overview for Stomach Adenocarcinoma (Primary Solid Tumor Cohort)—28 January 2016.

[22] Wickham H. (2016). Ggplot2—Elegant Graphics for Data Analysis.

[23] Pandya D., Mariani M., He S., Andreoli M., Spennato M., Dowell-Martino C., Fiedler P., Ferlini C. (2015). Epstein-Barr Virus MicroRNA Expression Increases Aggressiveness of Solid Malignancies. PLoS ONE.

[24] Sengupta S., den Boon J.A., Chen I.H., Newton M.A., Dahl D.B., Chen M., Cheng Y.J., Westra W.H., Chen C.J., Hildesheim A. (2006). Genome-wide expression profiling reveals EBV-associated inhibition of MHC class I expression in nasopharyngeal carcinoma. Cancer Res..

[25] Dutta N., Gupta A., Mazumder D.N., Banerjee S. (2006). Down-regulation of locus-specific human lymphocyte antigen class I expression in Epstein-Barr virus-associated gastric cancer: Implication for viral-induced immune evasion. Cancer.

[26] Truong C.D., Feng W., Li W., Khoury T., Li Q., Alrawi S., Yu Y., Xie K., Yao J., Tan D. (2009). Characteristics of Epstein-Barr virus-associated gastric cancer: A study of 235 cases at a comprehensive cancer center in U.S.A. J. Exp. Clin. Cancer Res..

[27] Kim S.Y., Park C., Kim H.J., Park J., Hwang J., Kim J.I., Choi M.G., Kim S., Kim K.M., Kang M.S. (2015). Deregulation of immune response genes in patients with Epstein-Barr virus-associated gastric cancer and outcomes. Gastroenterology.

[28] Boehm U., Klamp T., Groot M., Howard J.C. (1997). Cellular responses to interferon-gamma. Annu. Rev. Immunol..

[29] Becht E., Giraldo N.A., Lacroix L., Buttard B., Elarouci N., Petitprez F., Selves J., Laurent-Puig P., Sautes-Fridman C., Fridman W.H. (2016). Estimating the population abundance of tissue-infiltrating immune and stromal cell populations using gene expression. Genome Biol..

[30] Ohtani H., Jin Z., Takegawa S., Nakayama T., Yoshie O. (2009). Abundant expression of CXCL9 (MIG) by stromal cells that include dendritic cells and accumulation of CXCR3+ T cells in lymphocyte-rich gastric carcinoma. J. Pathol..

[31] Meissner T.B., Li A., Biswas A., Lee K.H., Liu Y.J., Bayir E., Iliopoulos D., van den Elsen P.J., Kobayashi K.S. (2010). NLR family member NLRC5 is a transcriptional regulator of MHC class I genes. Proc. Natl. Acad. Sci. USA.

[32] Meissner T.B., Liu Y.J., Lee K.H., Li A., Biswas A., van Eggermond M.C., van den Elsen P.J., Kobayashi K.S. (2012). NLRC5 cooperates with the RFX transcription factor complex to induce MHC class I gene expression. J. Immunol..

[33] Morrison T.E., Mauser A., Wong A., Ting J.P., Kenney S.C. (2001). Inhibition of IFN-gamma signaling by an Epstein-Barr virus immediate-early protein. Immunity.

[34] Shah K.M., Stewart S.E., Wei W., Woodman C.B., O’Neil J.D., Dawson C.W., Young L.S. (2009). The EBV-encoded latent membrane proteins, LMP2A and LMP2B, limit the actions of interferon by targeting interferon receptors for degradation. Oncogene.

[35] Hjalgrim H., Friborg J., Melbye M., Arvin A., Campadelli-Fiume G., Mocarski E., Moore P.S., Roizman B., Whitley R., Yamanishi K. (2007). The epidemiology of EBV and its association with malignant disease. Human Herpesviruses: Biology, Therapy, and Immunoprophylaxis.

[36] Hu L., Lin Z., Wu Y., Dong J., Zhao B., Cheng Y., Huang P., Xu L., Xia T., Xiong D. (2016). Comprehensive profiling of EBV gene expression in nasopharyngeal carcinoma through paired-end transcriptome sequencing. Front. Med..

[37] Hooykaas M.J., Kruse E., Wiertz E.J., Lebbink R.J. (2016). Comprehensive profiling of functional Epstein-Barr virus miRNA expression in human cell lines. BMC Genomics.

[38] Qiu J., Smith P., Leahy L., Thorley-Lawson D.A. (2015). The Epstein-Barr virus encoded BART miRNAs potentiate tumor growth in vivo. PLoS Pathog..

[39] Yang Y.C., Liem A., Lambert P.F., Sugden B. (2017). Dissecting the regulation of EBV’s BART miRNAs in carcinomas. Virology.

[40] Kouvidou C., Rontogianni D., Tzardi M., Datseris G., Panayiotides I., Darivianaki K., Karidi E., Delides G., Kanavaros P. (1995). Beta 2-microglobulin and HLA-DR expression in relation to the presence of Epstein-Barr virus in nasopharyngeal carcinomas. Pathobiology.

[41] Khanna R., Busson P., Burrows S.R., Raffoux C., Moss D.J., Nicholls J.M., Cooper L. (1998). Molecular characterization of antigen-processing function in nasopharyngeal carcinoma (NPC): Evidence for efficient presentation of Epstein-Barr virus cytotoxic T-cell epitopes by NPC cells. Cancer Res..

[42] Saiki Y., Ohtani H., Naito Y., Miyazawa M., Nagura H. (1996). Immunophenotypic characterization of Epstein-Barr virus-associated gastric carcinoma: Massive infiltration by proliferating CD8+ T-lymphocytes. Lab. Investig..

[43] Gong L.P., Chen J.N., Xiao L., He Q., Feng Z.Y., Zhang Z.G., Liu J.P., Wei H.B., Shao C.K. (2019). The implication of tumor-infiltrating lymphocytes in Epstein-Barr virus-associated gastric carcinoma. Hum. Pathol..

[44] Chakravorty S., Yan B., Wang C., Wang L., Quaid J.T., Lin C.F., Briggs S.D., Majumder J., Canaria D.A., Chauss D. (2019). Integrated pan-cancer map of EBV-associated neoplasms reveals functional host-virus interactions. Cancer Res..

[45] Moon J.W., Kong S.K., Kim B.S., Kim H.J., Lim H., Noh K., Kim Y., Choi J.W., Lee J.H., Kim Y.S. (2017). IFNgamma induces PD-L1 overexpression by JAK2/STAT1/IRF-1 signaling in EBV-positive gastric carcinoma. Sci. Rep..

[46] Sasaki S., Nishikawa J., Sakai K., Iizasa H., Yoshiyama H., Yanagihara M., Shuto T., Shimokuri K., Kanda T., Suehiro Y. (2019). EBV-associated gastric cancer evades T-cell immunity by PD-1/PD-L1 interactions. Gastric Cancer.

[47] Treece A.L., Duncan D.L., Tang W., Elmore S., Morgan D.R., Dominguez R.L., Speck O., Meyers M.O., Gulley M.L. (2016). Gastric adenocarcinoma microRNA profiles in fixed tissue and in plasma reveal cancer-associated and Epstein-Barr virus-related expression patterns. Lab. Investig..

[48] Hislop A.D., Ressing M.E., van Leeuwen D., Pudney V.A., Horst D., Koppers-Lalic D., Croft N.P., Neefjes J.J., Rickinson A.B., Wiertz E.J. (2007). A CD8+ T cell immune evasion protein specific to Epstein-Barr virus and its close relatives in Old World primates. J. Exp. Med..

[49] Zuo J., Quinn L.L., Tamblyn J., Thomas W.A., Feederle R., Delecluse H.J., Hislop A.D., Rowe M. (2011). The Epstein-Barr virus-encoded BILF1 protein modulates immune recognition of endogenously processed antigen by targeting major histocompatibility complex class I molecules trafficking on both the exocytic and endocytic pathways. J. Virol..

[50] Quinn L.L., Williams L.R., White C., Forrest C., Zuo J., Rowe M. (2016). The Missing Link in Epstein-Barr Virus Immune Evasion: The BDLF3 Gene Induces Ubiquitination and Downregulation of Major Histocompatibility Complex Class I (MHC-I) and MHC-II. J. Virol..

[51] Strong M.J., Laskow T., Nakhoul H., Blanchard E., Liu Y., Wang X., Baddoo M., Lin Z., Yin Q., Flemington E.K. (2015). Latent Expression of the Epstein-Barr Virus (EBV)-Encoded Major Histocompatibility Complex Class I TAP Inhibitor, BNLF2a, in EBV-Positive Gastric Carcinomas. J. Virol..

[52] Van Beek J., zur Hausen A., Snel S.N., Berkhof J., Kranenbarg E.K., van de Velde C.J., van den Brule A.J., Middeldorp J.M., Meijer C.J., Bloemena E. (2006). Morphological evidence of an activated cytotoxic T-cell infiltrate in EBV-positive gastric carcinoma preventing lymph node metastases. Am. J. Surg. Pathol..

[53] Park Y., Koh J., Kwak Y., Ahn S.H., Park D.J., Kim H.H., Kim W.H., Lee H.S. (2019). Clinicopathologic significance of human leukocyte antigen class I expression in patients with stage II and III gastric cancer. Cancer Immunol. Immunother..

[54] Spies T., Cerundolo V., Colonna M., Cresswell P., Townsend A., DeMars R. (1992). Presentation of viral antigen by MHC class I molecules is dependent on a putative peptide transporter heterodimer. Nature.

[55] Gameiro S.F., Zhang A., Ghasemi F., Barrett J.W., Nichols A.C., Mymryk J.S. (2017). Analysis of Class I Major Histocompatibility Complex Gene Transcription in Human Tumors Caused by Human Papillomavirus Infection. Viruses.

[56] Li H., Torabi S.J., Yarbrough W.G., Mehra S., Osborn H.A., Judson B. (2018). Association of Human Papillomavirus Status at Head and Neck Carcinoma Subsites With Overall Survival. JAMA Otolaryngol. Head Neck Surg..

